# Vemurafenib Limits Influenza A Virus Propagation by Targeting Multiple Signaling Pathways

**DOI:** 10.3389/fmicb.2017.02426

**Published:** 2017-12-14

**Authors:** Magdalena Holzberg, Yvonne Boergeling, Tobias Schräder, Stephan Ludwig, Christina Ehrhardt

**Affiliations:** ^1^Institute of Virology Muenster, Westfaelische Wilhelms-University Muenster, Muenster, Germany; ^2^Cluster of Excellence Cells in Motion, Westfaelische Wilhelms-University Muenster, Muenster, Germany

**Keywords:** Vemurafenib, Raf/MEK/ERK cascade, MAP kinases, influenza A virus, signal transduction pathways, apoptosis

## Abstract

Influenza A viruses (IAV) can cause severe global pandemic outbreaks. The currently licensed antiviral drugs are not very effective and prone to viral resistance. Thus, novel effective and broadly active drugs are urgently needed. We have identified the cellular Raf/MEK/ERK signaling cascade as crucial for IAV replication and suitable target for an antiviral intervention. Since this signaling cascade is aberrantly activated in many human cancers, several clinically approved inhibitors of Raf and MEK are now available. Here we explored the anti-IAV action of the licensed B-Raf^V600E^ inhibitor Vemurafenib. Treatment of B-Raf^WT^ cells with Vemurafenib induced a hyperactivation of the Raf/MEK/ERK cascade rather than inhibiting its activation upon IAV infection. Despite this hyperactivation, which has also been confirmed by others, Vemurafenib still strongly limited IAV-induced activation of other signaling cascades especially of p38 and JNK mitogen-activated protein kinase (MAPK) pathways. Most interestingly, Vemurafenib inhibited virus-induced apoptosis via impaired expression of apoptosis-inducing cytokines and led to hampered viral protein expression most likely due to the decreased activation of p38 and JNK MAPK. These multiple actions resulted in a profound and broadly active inhibition of viral replication, up to a titer reduction of three orders of a magnitude. Thus, while Vemurafenib did not act similar to MEK inhibitors, it displays strong antiviral properties via a distinct and multi-target mode of action.

## Introduction

Influenza virus infections can cause severe and life-threatening disease and annual epidemics result in enormous economic loss worldwide. An efficient way to protect against annual epidemics is vaccination. However, coverage is low and vaccinations may not confer protection against newly emerging influenza virus subtypes. Thus, efficient and broadly active antivirals are urgently needed, limiting viral replication in infected hosts (Antonelli and Turriziani, 2012).

Currently licensed therapeutics belong to two classes that either target viral neuraminidase or M2 ion channel function. A drawback of these antivirals is, that influenza viruses can rapidly gain resistances. Consequently, the use of M2 ion channel blockers for treatment of influenza virus infections is already contraindicated (World Health Organization, 2010). This demonstrates the medical need to develop novel antivirals against influenza viruses with a high barrier for resistance development that may be better achieved by targeting host factors rather than viral components (Maltezou and Tsiodras, 2009; Ludwig, 2011).

IAV modulate the activity of several cellular signaling cascades, which are in part exploited by the virus to enable efficient replication. These virus-supportive cellular signaling pathways came into focus as novel targets for antiviral therapies (Pleschka et al., 2001; Ludwig et al., 2003; Planz, 2013). One of these cascades is the Raf/MEK/ERK signaling cascade, which is important for the nuclear export of newly synthesized viral ribonucleoproteins (vRNPs) (Pleschka et al., 2001; Ludwig et al., 2004; Haasbach et al., 2017). In contrast to many other signaling events in IAV-infected cells, it is not activated by viral RNA (vRNA) sensing via RIG-I but rather is induced by accumulation of newly produced viral hemagglutinin (HA) in lipid rafts in the membrane of infected cells (Marjuki et al., 2006). Furthermore, it was recently shown that the presence of HA triggers the switch from MEK1 SUMOylation to an activating phosphorylation leading to the downstream activation of ERK1/2 (Wang et al., 2017). Thus, the cascade is implicated to control temporal coordination of virus particle assembly (Marjuki et al., 2006). Consequently, constitutive activation of the Raf/MEK/ERK signaling cascade was shown to support IAV replication (Olschlager et al., 2004), while its inhibition leads to decreased progeny virus titers (Pleschka et al., 2001; Ludwig et al., 2004; Droebner et al., 2011).

Remarkably, a hyperactivated Raf/MEK/ERK signaling cascade is causative for many human cancers, hence, some inhibitors directed against members of the cascade are already clinically approved for cancer therapy. One of these compounds is the B-Raf kinase inhibitor Vemurafenib, licensed as Zelboraf^TM^, which is used to treat malignant melanoma cells carrying an activating *BRAF* mutation of valine at position 600 (*BRAF*^*V600E*^) (Davies et al., 2002; Solit et al., 2006; Bollag et al., 2010, 2012; Flaherty et al., 2010; Chapman et al., 2011; Sosman et al., 2012). Inhibition of the mutated B-Raf kinase by Vemurafenib was shown to efficiently prevent activation of the Raf/MEK/ERK signaling cascade (Bollag et al., 2010, 2012; Flaherty et al., 2010; Chapman et al., 2011; Sosman et al., 2012). In contrast, it was also described to hyperactivate this cascade in *BRAF*^*WT*^ cells carrying oncogenic *RAS* or elevated upstream receptor signaling (Halaban et al., 2010; Vin et al., 2013) concomitant with high levels of off-target inhibition of various kinases including pathways necessary for efficient IAV replication (Pleschka et al., 2001; Ehrhardt et al., 2006; Nencioni et al., 2009; Nacken et al., 2012; Tahiri et al., 2013). Since Vemurafenib is already in clinical use for treatment of malignant melanoma with a well-characterized side-effect profile (Kim et al., 2014), the applicability of Vemurafenib for the treatment of IAV infections was elucidated *in vitro*.

The present study describes for the very first time a broad and strong anti-IAV activity of Vemurafenib against various IAV subtypes. Interestingly, Vemurafenib interferes with viral replication by inhibition of a multitude of different signaling cascades, which was independent of ERK1/2 activity. Thus, we provide evidence of a multi-target anti-IAV activity that encompasses a limitation of virus-induced apoptosis as well as a decrease in expression of viral proteins.

## Materials and methods

### Cell lines, viruses, and viral infections

Human lung epithelial cells (A549) were cultured in DMEM, Madin-Darby canine kidney epithelial cells (MDCK II) in MEM and human bronchial epithelial cells (Calu-3) in Ham's F-12/DMEM. Media were supplemented with 10% (v/v) heat-inactivated fetal bovine serum (FBS). Human IAV strains A/Puerto Rico/8/34 (H1N1, PR8M) and A/WSN/33 were obtained from the strain collection of the Institute of Virology Muenster (Germany). The avian IAV strain A/FPV/Bratislava/79 (H7N7, FPV) was originally taken from the strain repository of the Institute of Virology, Giessen, Germany. Mouse-adapted A/Seal/Massachusetts/1/80 (H7N7, SC35M) (described in Gabriel et al., 2005), and A/Anhui/1/2013 (H7N9) were a kind gift from Thorsten Wolff, Robert-Koch-Institute (Berlin, Germany). All experiments and handling of samples containing H7N9 or H7N7 FPV infectious particles were performed in a biological safety level 3 containment. All viruses were propagated on MDCK II cells and infections were performed as described previously (Hrincius et al., 2011).

### Reagents and stimulation of cells

Vemurafenib (PLX-4032, Zelboraf™) was purchased from Active Biochem and U0126 was taken from the inhibitor stock of the Institute of Virology, Muenster (Germany). SB202190 and SP600125 were purchased from Calbiochem.

For stimulation with human epidermal growth factor (EGF) or human tumor necrosis factor alpha (TNFα), A549 cells were pretreated with Vemurafenib (25 μM) or DMSO in DMEM for 60 min. Subsequently, EGF (R&D Systems) (30 ng/ml) or TNFα (Sigma-Aldrich) (5 ng/ml) were added to the medium and cells were lysed after 5 min (EGF) or 30 min (TNFα), respectively.

Apoptosis was induced by treatment of A549 cells with 1 μM Staurosporine (Sigma-Aldrich) for 5 h, with human TNFα (5 ng/ml) (Sigma-Aldrich) or with human Super*Killer*TRAIL™ (50 ng/ml) (Enzo Life Sciences GmbH) for 4 h.

For viral RNA stimulation, total RNA was isolated from mock-infected (cellular RNA, cRNA) or FPV-infected (MOI 5; viral RNA, vRNA) A549 cells 8 hours post infection (hpi) using peqGOLD TriFast™ (Peqlab) reagent as described previously (Börgeling et al., 2014).

For co-treatment of A549 cells with U0126 and Vemurafenib, cells were pretreated with U0126 (50 μM) for 30 min before Vemurafenib (25 μM) was added. After 60 min, cells were transfected with 500 ng vRNA or cRNA per 12-well using Lipofectamine® 2000 (Thermo Fisher Scientific). Six hours post transfection, cell lysates were subjected to reverse transcription followed by qRT-PCR.

### Standard plaque titration

Plaque titration was performed as described earlier (Seyer et al., 2012). Plaques were visualized by neutral red staining and progeny virus titers are depicted as plaque forming units per milliliter (PFU/ml).

### Cell proliferation assay (WST-1)

A549 cells were grown in 96-well plates and cultured in presence of Vemurafenib or DMSO. Staurosporine (Sigma-Aldrich) (2 μM) was added to the medium as positive control. At the times indicated, WST-1 (water soluble tetrazolium)-based cell proliferation assay (Roche) was performed according to the manufacturer's instructions. Absorbance was measured at 450 nm.

### Reverse transcription and quantitative real-time PCR (qRT-PCR*)*

Total RNA was isolated using the RNeasy Mini Kit (Qiagen) as described by the manufacturer. Subsequently, 500 ng total RNA was reverse transcribed with RevertAid H Minus ReverseTranscriptase (Thermo Fisher Scientific) and oligo(dT) primers (MWG-Biotech AG) according to the manufacturer's protocol. The cDNA was subjected to qRT-PCR, which was performed using a Roche LightCycler 480 and Brilliant SYBR Green III Mastermix (Agilent) according to the manufacturer's instructions. The following primers were used: human glyceraldehyde 3-phosphate dehydrogenase (GAPDH) forward (5′-gcaaattccatggcaccgt-3′) and reverse (5′-gccccacttgatttggagg-3′), IAV polymerase basic protein 1 (PB1) forward (5′-catacagaagaccagtcgggat-3′) and reverse (5′-gtctgagctcttcaatgtggtgga-3′); IAV matrix protein 1 (M1) forward (5′-tgcaaaaacatcttcaagtctctg-3′) and reverse (5′-agatgagtcttctaaccgaggtcg-3′), IAV non-structural protein (NS) forward (5′-gaggacttgaatggaatgataaca-3′) and reverse (5′-gtctcaattcttcaatcaatcaaccatc-3′), human tumor necrosis factor-related apoptosis-inducing ligand (TRAIL) forward (5′-gtctctctgtgtggctgtaacttacg-3′) and reverse (5′-aaacaagcaatgccacttttgg-3′) and human tumor necrosis factor alpha (TNFα) forward (5′-atgagcactgaaagcatgatc-3′) and reverse (5′-gagggctgattagagagaggt-3′). Relative changes in expression levels (*n*-fold) were determined by the 2^ΔΔ*Ct*^ method (Livak and Schmittgen, 2001).

### Western blot analysis

For western blot analysis, cells were lysed in radioimmunoprecipitation assay buffer (RIPA) as described earlier (Seyer et al., 2012). Protein lysates were cleared by centrifugation, mixed with 5x Laemmli buffer, separated by SDS-PAGE and blotted onto nitrocellulose membranes.

Antisera directed against ERK2 (C-14; #sc-154) and IAV PB1 (vK-20; #sc-17601) were purchased from Santa Cruz Biotechnology and α-Tubulin antibodies (#T6199) from Sigma-Aldrich. Antiserum against IAV PB2 protein was a kind gift of E. Fodor (Sir William Dunn School of Pathology, Oxford, UK; Carr et al., 2006). Mouse monoclonal antibodies against IAV NS1 were developed at the Institute of Virology Muenster (Germany) and can be purchased from Santa Cruz Biotechnology (#sc-130568). IAV M1 (#MCA-401) antibodies were obtained from AbD Serotec, while antibodies directed against IAV M2 (#GTX125951) and NS2/NEP (#GTX125953) were purchased from Genetex.

Antibodies directed against the phospho-sites of Akt (Ser473; #9271), ATF2 (Thr71; #9221), ERK1/2 (p44/42 MAPK, Thr202/Tyr204; #9106), MK2 (Thr222; #3316), MEK1/2 (Ser217/221, 41G9; #9154), 4E-BP1 (Thr37/46, 236B4; #2855), p-p70S6K (Thr389, 108D2; #9234), S6 Rib. Prot. (Ser235/236, D57.2.2E; #4858), MKK3/MKK6 (Ser189/Ser207, 22A8; #9236) as well as antibodies for detection of Caspase 3 (#9662), Caspase 8 (cleaved, 18C8, Asp391; #9496), Caspase 8 (D35G2; #4790), Caspase 9 (cleaved, Asp315; #9505), and Caspase 9 (C9; #9508) were obtained from Cell Signaling Technology. Phospho-specific antibodies directed against JNK (Thr183/Tyr185; #612541) and p38 MAPK (Thr180/Tyr182; #612281) and antiserum directed against PARP (#611039) were purchased from BD Bioscience.

### Immunofluorescence staining

A549 cells infected with FPV (MOI 5) were fixed with 4% formaldehyde for 15 min at 4°C at the indicated time points. After washing, permeabilization was performed with 0.1% TritonX-100 for 15 min at RT. Nucleoprotein (NP) localization was detected using anti-influenza A virus NP primary antibodies (AbD Serotec, #MCA-400) and Alexa Fluor® 488 chicken anti-mouse IgG secondary antibodies (Thermo Fisher Scientific, #A21200). Nuclei were counterstained with DAPI (Thermo Fisher Scientific). For quantification, two random areas were analyzed using the fluorescence microscope BIOZERO (Keyence). Quantification of nuclear localization was performed using FIJI Software's Cell Counter plugin (Schindelin et al., 2012).

### Statistical analysis

Statistical significance was calculated with GraphPad Prism software versions 5 and 6 using the indicated statistical tests. *P*-values are indicated by asterisks ^*^p = 0.01–0.05; ^**^p = 0.001–0.01; ^***^p = 0.0001–0.001; ^****^p < 0.0001.

## Results

### Vemurafenib efficiently impairs influenza a virus replication independent of cytostatic activities

**Figure 1:**
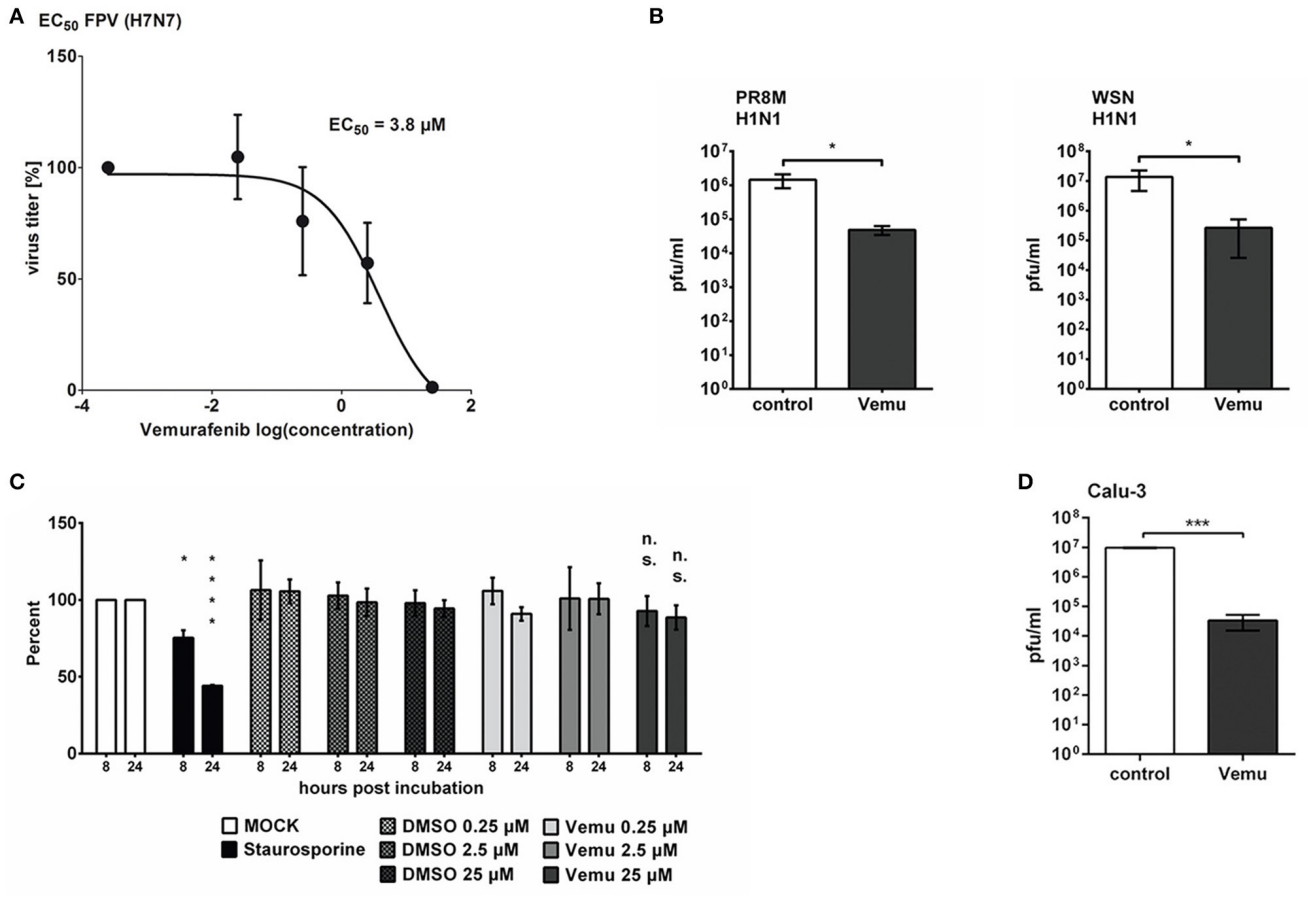
Vemurafenib inhibits IAV replication in A549 cells. **(A)** Determination of the effective concentration 50% (EC_50_) of Vemurafenib in A549 cells infected with MOI 0.01 H7N7 (FPV). Cells were subsequently treated with different concentrations of Vemurafenib (0–25 μM). Progeny virus particles in the supernatant were measured by standard plaque assay 24 hpi. The EC_50_ values were calculated from three independent experiments with GraphPad Prism 5 software and depicted as mean (±SEM). **(B)** A549 cells were treated with Vemurafenib (25 μM) or DMSO after infection with 0.1 MOI human IAV H1N1 subtypes PR8M (left panel) or WSN (right panel). Infectious particles in the supernatant were determined by standard plaque assay 24 hpi. Shown are means (±*SD*) of at least three independent experiments. Statistical significance was evaluated by unpaired, two-tailed *t*-test (^*^p = 0.01–0.05). **(C)** Vemurafenib-induced changes in A549 cell proliferation were analyzed by WST-1 assay (Roche). Shown are means (±*SD*) of three independent experiments normalized to MOCK. Statistical significance of differences to MOCK was evaluated by two-way ANOVA followed by Sidak's multiple comparisons test (^*^p = 0.01–0.05; ^****^p < 0.0001). **(D)** Calu-3 cells were infected with 0.01 MOI of SC35M and subsequently treated with 25 μM Vemurafenib or DMSO. Progeny virus particles in the supernatant were measured by standard plaque assay 24 hpi. Shown are means (±*SD*) of one representative out of three independent experiments. Statistical significance was analyzed by unpaired, two-tailed *t*-test (^***^p = 0.0001–0.001).

Inhibition of the Raf/MEK/ERK signaling cascade has been shown to limit the replication of various influenza viruses based on the retention of viral ribonucleoprotein complexes in the nucleus (Pleschka et al., 2001). But, so far, nothing is known about the potential influence of the clinically approved B-Raf^V600E^ inhibitor Vemurafenib on viral propagation. Therefore, wildtype *BRAF*-containing human lung epithelial A549 cells were treated with the indicated concentrations of Vemurafenib and were subsequently infected with the IAV strain A/FPV/Bratislava/79 (H7N7, FPV). The effective concentration 50% (EC_50_) was analyzed by standard plaque assays 24 hpi. Here, Vemurafenib efficiently inhibited viral replication already at low micromolar concentrations (EC_50_ = 3.8 μM; Figure 1A). Furthermore, strain-specific differences in EC_50_ were observed, with the compound being most effective in SC35M infection [SC35M (H7N7): 0.22 μM; Figure S1A]. Of note, Vemurafenib was also active against the recently emerged viruses of the H7N9 subtype with a quite low EC_50_ [A/Anhui (H7N9): 0.64 μM; Figure S1B]. Nevertheless, for all strains tested, the most efficient decrease in progeny virus titers was observed with concentrations higher than 20 μM, leading to a reduction of viral replication of more than one log step. Next, the question arises as to whether this effect is specific for avian influenza viruses or whether the presence of Vemurafenib decreases viral replication of IAV strains of human subtypes as well. As expected, the antiviral activity of Vemurafenib against avian IAV was confirmed for human IAV. The human H1N1 strains A/Puerto Rico/8/34 (PR8M) and A/WSN/33 (WSN) were also highly sensitive to Vemurafenib (Figure 1B), indicating a broad antiviral activity against various IAV strains of different origin.

The Raf/MEK/ERK signaling cascade is known to transmit signals from mitogen- and growth factor-receptors to regulate cell proliferation and apoptosis (Mebratu and Tesfaigzi, 2009), cellular processes that have been shown to be involved in efficient IAV replication (Wurzer et al., 2003; He et al., 2010). To exclude that the described effect of Vemurafenib on viral propagation is due to impaired cell viability, WST-1-based proliferation assays were performed. As cleavage of the substrate WST-1 only occurs in living and metabolically active cells, cellular proliferation and viability can be determined. The results shown in Figure 1C clearly indicate that the inhibitor did not significantly affect cellular proliferation and viability. Thus, the observed impact of Vemurafenib on viral replication occurs independent of modulated cell viability. A549 cells have been shown to possess special features, e.g. a distinct mode of apoptosis induction (Hasegawa et al., 2007). To exclude that Vemurafenib-mediated effects on viral replication are based on cell line specific features, other susceptible cells, such as the human bronchial epithelial Calu-3 cells, were infected with the IAV strain SC35M and subsequently treated with Vemurafenib. Also here, Vemurafenib efficiently inhibited viral replication (Figure 1D), ruling out that the observed effect is cell line specific.

In summary, Vemurafenib possesses a prominent and broad antiviral activity, significantly impairing replication of different IAV subtypes and strains that is independent of cytostatic or toxic activities.

### Vemurafenib hyperactivates the Raf/MEK/ERK pathway but inhibits general MAPK signaling

Vemurafenib inhibits B-Raf kinase activity in cells carrying *BRAF*^*V600E*^, nevertheless, it was described to hyperactivate the Raf/MEK/ERK signaling cascade in *BRAF*^*WT*^ cells (Halaban et al., 2010; Vin et al., 2013). Furthermore, Vemurafenib has been shown to possess high levels of off-target inhibition of various kinases in exposure of *ex-vivo* melanoma tumor lysates (Tahiri et al., 2013). Interestingly, here, the kinase substrates that distinguished between *BRAF*^*WT*^ and *BRAF*^*V600E*^ tumors represented kinases mainly involved in the phosphatidylinositide 3-kinase (PI3K) and MAPK signaling network. Importantly, these signal transduction pathways were described to be necessary for efficient IAV replication (Pleschka et al., 2001; Ehrhardt et al., 2006; Nencioni et al., 2009; Nacken et al., 2012).

**Figure 2:**
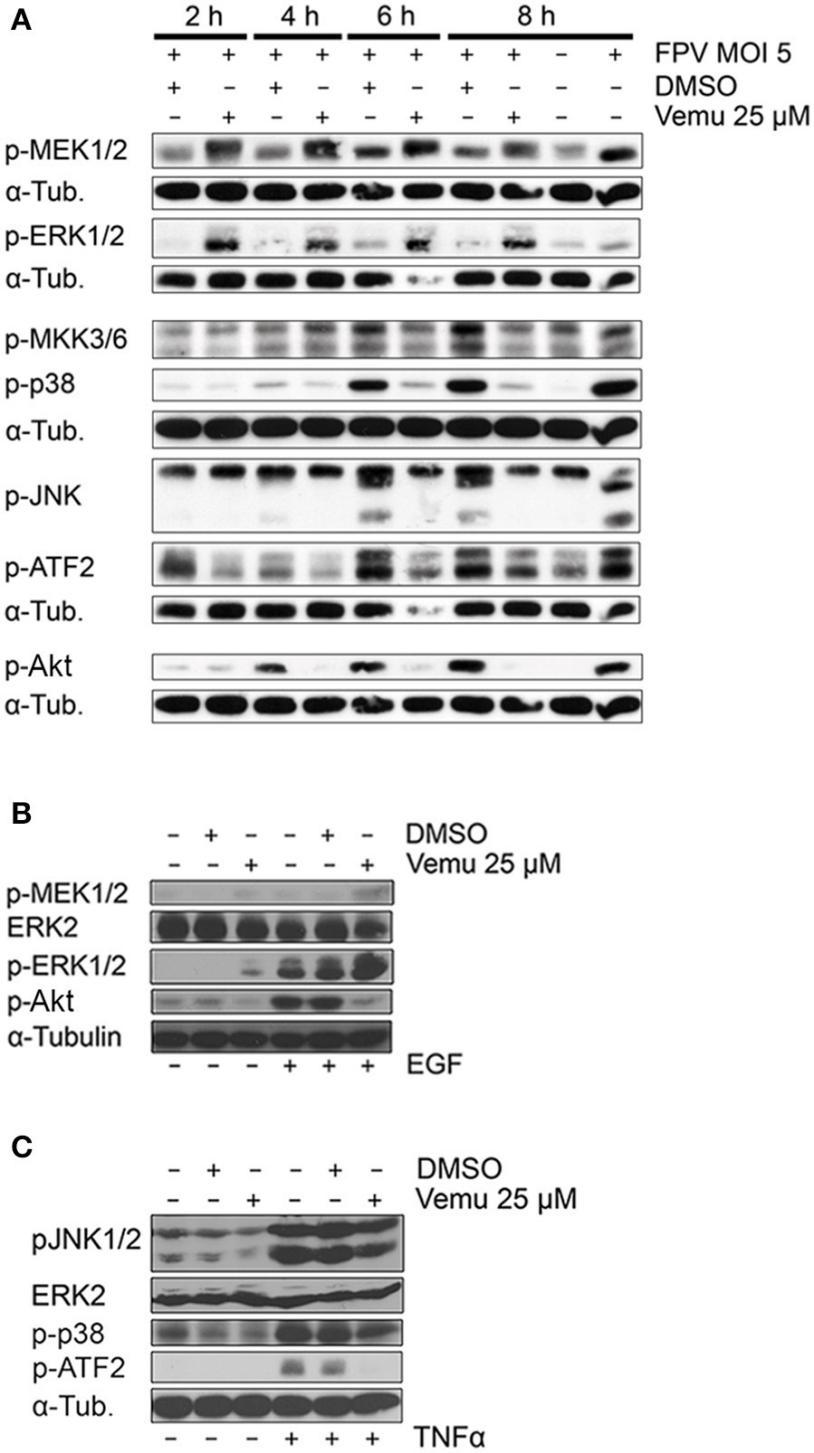
Vemurafenib hyperactivates the Raf/MEK/ERK pathway while inhibiting various cellular signaling cascades. **(A)** Analysis of the effects of Vemurafenib on IAV-induced cellular signaling pathways by western blot. A549 cells were infected with FPV (MOI 5) and subsequently treated with Vemurafenib (25 μM) or DMSO, respectively. Total cell lysates were harvested at the times indicated. Activity of the Raf/MEK/ERK pathway was analyzed by detection of phosphorylated kinases MEK1/2 and ERK1/2. JNK and p38 MAPK pathway activities were elucidated by analysis of JNK1/2 and p38 phosphorylation as well as by detection of phosphorylation of upstream kinases MKK3/6 and downstream target ATF2. Activity of the PI3K/Akt pathway was determined by detection of Akt phosphorylation. Alpha-tubulin served as loading control. **(B,C)** Stimulation of Vemurafenib-treated A549 cells with EGF (30 ng/ml) for 5 min **(B)** or TNFα (5 ng/ml) for 30 min **(C)**. Activity of different cellular signaling pathways was analyzed by western blot. ERK2 and alpha-tubulin served as loading controls. **(A–C)** Blots are representative of three independent experiments.

To decipher signaling cascades involved in Vemurafenib-mediated inhibition of viral replication, the impact of the compound on IAV-induced cellular signaling cascades was analyzed in *BRAF*^*WT*^-carrying A549 cells. Indeed, Vemurafenib caused a hyperinduction of the Raf/MEK/ERK signaling cascade as shown by increased phosphorylation of ERK1/2 as well as MEK1/2 (Figure 2A). In contrast, IAV-induced activation of all other cellular signaling cascades analyzed, such as MAPKs JNK and p38 but also the PI3K/Akt pathway, were strongly inhibited by Vemurafenib treatment (Figure 2A).

To exclude that the reduced activation of cellular signaling cascades in presence of Vemurafenib is only secondary due to altered viral replication, induction of signal transduction was analyzed in a virus-free approach. A549 cells were pretreated with Vemurafenib and subsequently stimulated with epidermal growth factor (EGF), a well-known inducer of the Raf/MEK/ERK signaling cascade (Figure 2B). Here, Vemurafenib effectively inhibited EGF-induced PI3K signaling as demonstrated by a decreased phosphorylation of the Akt kinase, while still hyperactivating the Raf/MEK/ERK pathway. To analyze whether Vemurafenib also inhibits proinflammatory and stress-related signaling, A549 cells were stimulated with tumor necrosis factor alpha (TNFα). Interestingly, Vemurafenib treatment significantly inhibited TNFα-activated JNK and p38 MAPK signaling cascades resulting in a decreased phosphorylation of downstream transcription factor ATF2 (Figure 2C), highlighting that Vemurafenib directly limits the activation of various cellular signaling cascades while inducing a hyperactivation of the Raf/MEK/ERK signaling cascade. This broad interference with the activity of cellular kinases is not a secondary effect caused by impaired virus replication and might contribute to the antiviral potential of Vemurafenib.

### Vemurafenib inhibits apoptosis onset by suppressing IAV-induced TRAIL expression

Besides a hyperactivation of the Raf/MEK/ERK signaling cascade and decreased cellular kinase activation, Vemurafenib has been shown to suppress apoptosis as a result of reduced MAPK JNK signaling *in vitro* and *in vivo* (Vin et al., 2013). Upon IAV infection, apoptosis is initiated by multiple viral factors resulting in the expression of apoptosis-inducing cytokines such as TRAIL (TNF-related apoptosis-inducing ligand) (Lowy, 2003), which lead to the cleavage of initiator caspases 8 and 9 and activate downstream executioner caspase 3 (Igney and Krammer, 2002). Importantly, proper timely regulation of apoptosis was shown to be crucial for efficient IAV replication by contributing to the nuclear export of progeny vRNPs (Wurzer et al., 2003; Muhlbauer et al., 2015).

**Figure 3:**
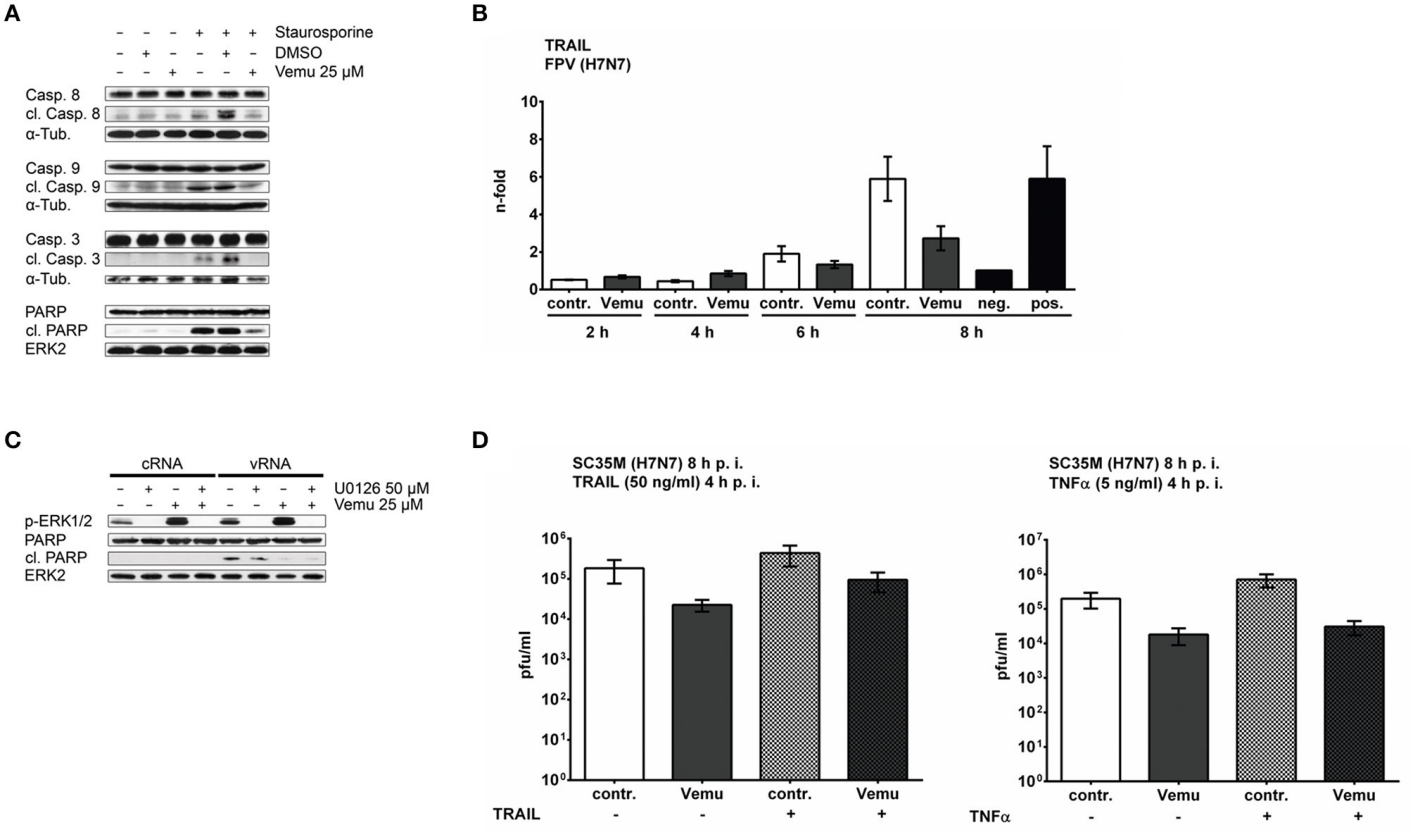
Vemurafenib inhibits IAV-induced apoptosis by interfering with cytokine expression. **(A)** A549 cells were treated with Vemurafenib (25 μM), DMSO or left untreated and were stimulated with Staurosporine (1 μM) for 5 h. Cleavage of caspases 8, 9, and 3 as well as PARP was visualized by western blot of total cell lysates. ERK2 and alpha-tubulin served as loading controls. Blots are representative of three independent experiments. **(B)** Expression levels of TRAIL mRNA in FPV-infected (MOI 5) A549 cells which were subsequently treated with Vemurafenib (25 μM) or DMSO, respectively. Untreated samples served as negative (uninfected) and positive control (infected). Results are depicted as mean *n*-fold expression (±SEM) of three independent experiments normalized to negative control. Data were analyzed for statistical significance by Kruskal–Wallis test followed by Dunn's multiple comparisons test. **(C)** A549 cells were pre-incubated with U0126 (50 μM) for 90 min and Vemurafenib (25 μM) for 60 min or DMSO before transfection of 500 ng total RNA isolated from infected A549 cells (vRNA; FPV, MOI 5, 8 h). Total RNA from uninfected cells (cRNA) was used as control. Phosphorylation of ERK1/2 was confirmed by immunostaining with phospho-specific antibodies. Apoptosis induction was analyzed by detection of cleaved PARP. Equal loading was confirmed by staining of ERK2. Blots are representative of three independent experiments. **(D)** A549 cells were infected with H7N7 (SC35M) and afterwards treated with Vemurafenib (25 μM) or DMSO, respectively. Cells were stimulated with human TRAIL (50 ng/ml) (left panel) or human TNFα (5 ng/ml) (right panel) 4 hpi. Infectious virus particles in the supernatant were measured 8 hpi by standard plaque assay and are depicted as mean (±*SD*) of three independent experiments. Statistical significance was analyzed by one way ANOVA followed by Sidak's multiple comparisons test.

To analyze the effects of Vemurafenib on apoptosis induction, A549 cells were treated with Vemurafenib or DMSO and were subsequently stimulated with Staurosporine, a potent inducer of apoptosis. As marker for apoptosis progression, cleavage of caspases 8, 9, and 3 as well as of the downstream acting poly (ADP-ribose) polymerase (PARP) was investigated by western blot analysis. As expected, Vemurafenib readily inhibited Staurosporine-induced apoptosis as demonstrated by decreased PARP cleavage (Figure 3A). Remarkably, not only activation of caspases 3 and 8 belonging to the TRAIL-induced so-called extrinsic pathway were inhibited, but also activation of caspase 9 of the intrinsic pathway was strongly decreased. These results highlight an early inhibition of Staurosporine-mediated apoptosis induction by Vemurafenib occurring upstream of caspase activation.

It was previously shown that IAV initiates apoptosis in late phases of viral replication by induction of different cellular signaling cascades leading to the expression of TRAIL, TNFα, or Fas ligand (Wurzer et al., 2004). Furthermore, it has been described that MAPKs such as JNK as well as p38 are involved in the IAV-induced expression of apoptosis-inducing cytokines (Maruoka et al., 2003; Lee et al., 2005). Both are signaling cascades that are efficiently inhibited by Vemurafenib. To answer the question as to whether the compound interferes with IAV-mediated expression of apoptosis-inducing cytokines, A549 cells were pretreated with Vemurafenib or DMSO and were subsequently infected with FPV. TRAIL mRNA expression was analyzed by qRT-PCR. As expected, IAV infection resulted in the expression of TRAIL mRNA starting at 6 hpi, which was efficiently inhibited by Vemurafenib treatment (Figure 3B). To conclude, Vemurafenib inhibits IAV-mediated apoptosis already early at the level of apoptosis-inducing cytokine expression.

The activity of MAPKs is known to be closely related to the regulation of apoptosis and, in context of IAV infection, pro- as well as anti-apoptotic functions have been described (Wada and Penninger, 2004). Particularly, the Raf/MEK/ERK signaling cascade, which is hyperactivated by Vemurafenib treatment, has been shown to exert anti-apoptotic as well as proliferation promoting functions (Mebratu and Tesfaigzi, 2009). To analyze whether the Vemurafenib-induced inhibition of apoptosis during IAV infection results from the hyperactivation of the Raf/MEK/ERK signaling cascade, ERK1/2 activation was inhibited by pretreatment with the MEK inhibitor U0126. To avoid differences in apoptosis progression due to altered viral replication, a non-dynamic stimulus mimicking viral infection was used. The main pathogen-associated molecular pattern (PAMP), that is sensed by different pattern-recognition receptors (PRRs) to induce cellular signaling cascades in viral infections, is viral RNA. Particularly, detection of the 5′-triphosphate structure in viral RNAs by the cytoplasmic helicase RIG-I plays an important role in IAV infection (Pichlmair et al., 2006). Therefore, A549 cells were transfected with total RNA isolated from virus-infected cells (vRNA) or from uninfected cells (cRNA) as a control. Cleavage of PARP was used as indicator for onset of apoptosis. Interestingly, transfection of RNA led to an induction of ERK phosphorylation, which was efficiently blocked by the MEK inhibitor U0126 (Figure 3C). Furthermore, presence of Vemurafenib enhanced ERK1/2 activation while there was no phosphorylation detectable in combinational treatment with U0126. Interestingly, while the stimulation with vRNA- induced apoptosis, which was not affected by MEK inhibition, Vemurafenib significantly inhibited PARP cleavage even in presence of U0126. Thus, Vemurafenib-induced hyperactivation of the Raf/MEK/ERK signaling cascade is not responsible for the interference with IAV-induced apoptosis. Furthermore, these results were verified on the Vemurafenib-mediated inhibition of IAV-induced TRAIL as well as TNFα mRNA expression, showing no significant effects of inhibition of ERK1/2 hyperactivation (Figure S2). Therefore, Vemurafenib inhibits IAV-mediated apoptosis-inducing cytokine expression independent of ERK1/2 hyperactivation, suggesting an important role of the inhibition of different stress kinase pathways by Vemurafenib resulting in the decreased expression of apoptosis-inducing cytokines.

It has been shown previously that induction of apoptosis in infected cells is required for efficient viral replication, and that stimulation with TRAIL consequently enhances viral propagation (Wurzer et al., 2004). To analyze whether the anti-viral effect of Vemurafenib is mediated by inhibition of cytokine-induced apoptosis, A549 cells were infected with SC35M and were treated with Vemurafenib or DMSO. Subsequently, cells were stimulated with recombinant TRAIL or TNFα and progeny virus titers were analyzed 8 hpi. As expected, treatment with Vemurafenib resulted in reduced progeny virus titers (Figure 3D). Furthermore, stimulation with TRAIL as well as with TNFα showed the tendency to enhance viral replication. Surprisingly, this tendency was also observed in Vemurafenib-treated cells in presence of TRAIL, but not with TNFα stimulation. Nevertheless, TRAIL stimulation was not sufficient to completely rescue viral replication.

In summary, Vemurafenib strongly impairs viral replication, an effect that is partially attributed to the inhibition of IAV-induced apoptosis mediated by reduced TRAIL expression.

### Vemurafenib interferes with the expression of viral proteins

While Vemurafenib targets the Raf/MEK/ERK signaling cascade causing its hyperactivation, IAV-induced p38 and JNK MAPK signaling as well as apoptosis onset were efficiently inhibited. These Vemurafenib-affected pathways were all described to have an impact on viral propagation (Ludwig et al., 2006; Ehrhardt and Ludwig, 2009). To gain further insights in Vemurafenib-mediated inhibition of viral replication, different steps in viral life cycle progression were analyzed.

Inhibition of the Raf/MEK/ERK signaling cascade has been shown to impair viral replication by nuclear retention of progeny vRNPs (Pleschka et al., 2001). Furthermore, Nencioni and colleagues described the same retention of vRNPs when p38 MAPK was inhibited in Madin-Darby canine kidney cells, and IAV-induced apoptosis progression has been shown to be required for RNP export in late stages of the replication cycle as well (Wurzer et al., 2003; Muhlbauer et al., 2015). So far, nothing is known about the interplay of these different pathways in nuclear RNP export and whether this plays a role in Vemurafenib-mediated interference with viral replication.

**Figure 4:**
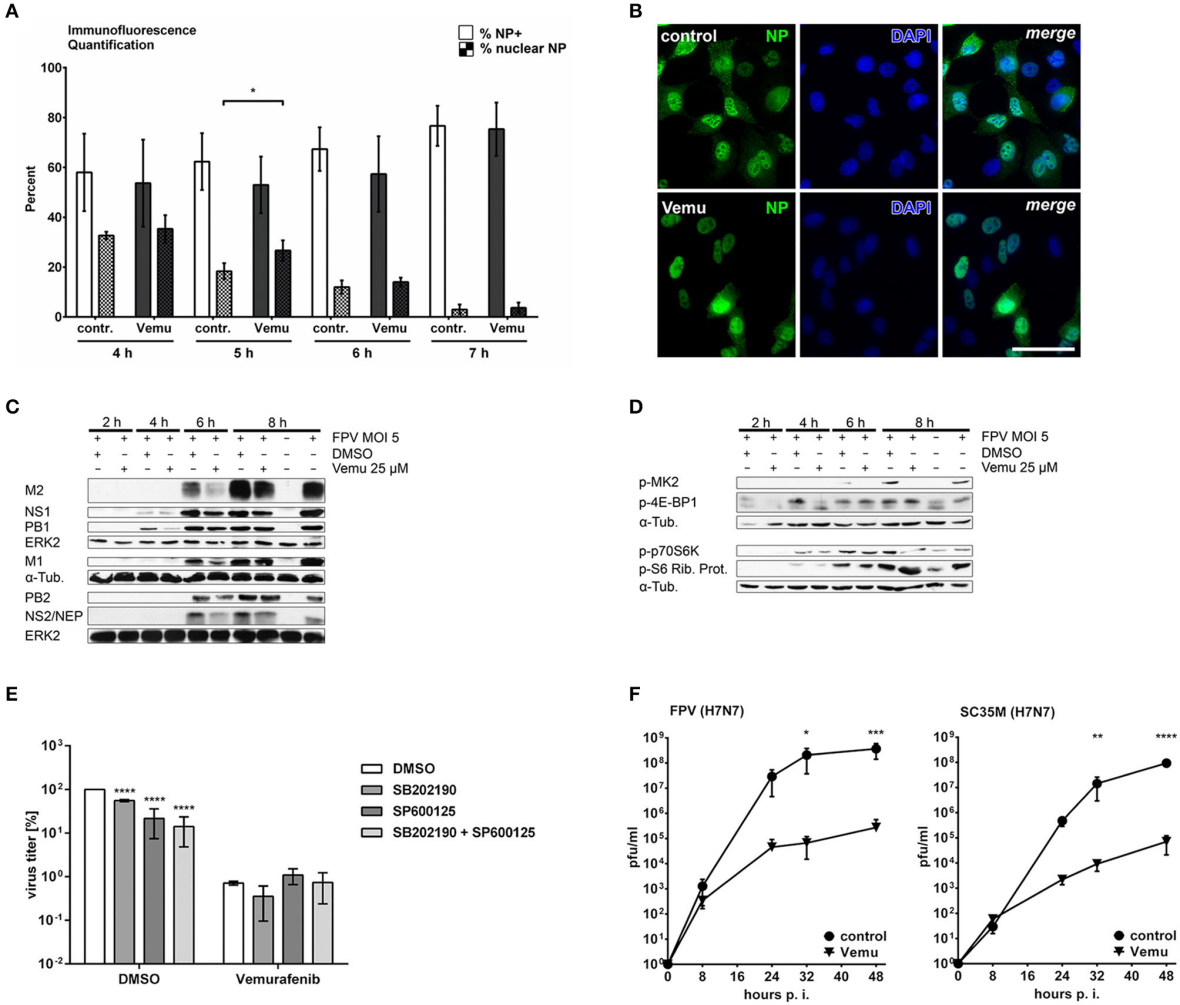
Vemurafenib strongly limits influenza A virus replication by a multifactorial mode of action. **(A,B)** Immunofluorescence of Vemurafenib (25 μM)- or DMSO-treated A549 cells infected with FPV (MOI 5) for the time points indicated. Viral NP localization was detected using IAV NP primary antibodies and nuclei were counterstained with DAPI. **(A)** NP positive cells (open bars) and cells with nuclear NP localization (checkered bars) were quantified and related to the number of DAPI positive cells. FIJI software was used for counting. Statistical significance of % NP positive cells and % nuclear NP was evaluated separately by one-way ANOVA followed by Sidak's multiple comparisons test (^*^p = 0.01–0.05). **(B)** Exemplary sections of one of three independent experiments. Scale represents 50 μm. **(C,D)** A549 cells were infected with FPV (MOI 5) and afterwards treated with Vemurafenib (25 μM) or DMSO, respectively. **(C)** Expression of viral proteins was analyzed by western blot assay at the times indicated. ERK2 and alpha-tubulin served as loading controls. **(D)** Samples were further analyzed for phosphorylation levels of MK2, 4E-BP1, p70S6K, and pS6 Rib. Prot. by western blot. Alpha-Tubulin served as loading control. **(C,D)** Blots are representative of three independent experiments. **(E)** A549 cells were infected with 0.01 MOI SC35M and subsequently treated with 10 μM SB202190, 10 μM SP600125 or both inhibitors in presence of Vemurafenib (25 μM) or DMSO, respectively. Amounts of progeny virions were measured by standard plaque assay. Shown are means (±*SD*) of three independent experiments. Statistical significance was calculated by two-way analysis of variance (ANOVA) followed by Dunnett's multiple comparisons test relative to respective DMSO controls (^****^p < 0.0001). **(F)** A549 cells were infected with 0.01 MOI avian IAV subtypes FPV (left panel) or SC35M (right panel) and subsequently treated with Vemurafenib (25 μM) or DMSO, respectively. Shown are means (±*SD*) of three independent experiments measured by standard plaque assay. Statistical significance was calculated by two-way analysis of variance (ANOVA) followed by Sidak's multiple comparisons test (^*^p = 0.01–0.05; ^**^p = 0.001–0.01; ^***^p = 0.0001–0.001; ^****^p < 0.0001).

For this reason, intracellular localization of vRNPs was analyzed by detection of nucleoprotein (NP), which is the major component of vRNP complexes. Importantly, Vemurafenib did not alter the rate of infected cells as the amount of NP positive cells did not change due to Vemurafenib treatment (% NP+, open bars), indicating that there is no obvious impact of the kinase inhibitor on virus entry (Figure 4A). While Vemurafenib slightly and transiently delayed nuclear vRNP export at 5 hpi (Figure 4A, % nuclear NP, checkered bars and Figure 4B), progeny vRNPs were readily exported from the nucleus at later time points compared to DMSO-treated cells. These results indicate that in presence of Vemurafenib, active nuclear vRNP export mediated by the hyperactivated Raf/MEK/ERK signaling cascade might compensate for the decreased passive export attributed to apoptosis inhibition.

Since impaired nuclear vRNP export does not seem to be the cause for reduced viral replication in Vemurafenib-treated cells, the mechanistic background of Vemurafenib action was further explored by addressing other important steps in the viral life cycle, namely transcription and translation. A549 cells were pretreated with Vemurafenib or DMSO and subsequently infected with FPV. After cell lysis, viral protein expression was investigated by western blot analysis (Figure 4C). Here, expression of several viral proteins was reduced in presence of Vemurafenib already early upon infection, notably affecting proteins expressed from spliced RNAs such as M2 and NEP. Interestingly, treatment with Vemurafenib did not significantly alter the expression of viral PB1, NS, or M1 m/cRNAs at all time points analyzed (Figure S3), indicating a direct impact of Vemurafenib on viral protein translation.

Since expression of viral proteins depends on the host cell translation machinery, several important cellular proteins involved in translation were analyzed for their activation. Here, analysis was focused on downstream targets of the signaling pathways that have been previously shown to be inhibited by Vemurafenib. MAPK-activated protein kinase-2 (MAPKAPK2, MK2) has been shown to be activated by MAPK p38 in IAV infection and to play a role in the inhibition of protein kinase R (PKR) (Luig et al., 2010), a kinase that represses cellular protein translation thereby limiting viral replication. As expected, IAV-induced MK2 phosphorylation was strongly inhibited by Vemurafenib (Figure 4D). On the contrary, the eukaryotic translation initiation factor 4E-binding protein 1 (4E-BP1) was affected in Vemurafenib-treated cells only 4 hpi. 4E-BP1 is a repressor of translation initiation that gets inactivated by phosphorylation, which is induced by the PI3K pathway (Gingras et al., 1998). Furthermore, there were no considerable differences in the activation of ribosomal protein S6 kinase beta-1 (p70S6K) and its downstream target ribosomal protein S6 (S6 Rib. Prot.) at all time points analyzed. The latter proteins are described to be activated by PI3K signaling leading to an induction of protein synthesis (Chung et al., 1994). Together, these results indicate that viral protein synthesis but not viral transcription is affected by Vemurafenib, possibly due to an inhibition of MK2 activation leading to a more pronounced PKR-mediated translation inhibition.

Taken together, Vemurafenib-induced inhibition of viral replication is based on impaired MAPK-mediated cellular functions such as induction of apoptosis and protein expression. To analyze whether MAPKs p38 and JNK are involved in the observed antiviral effects of Vemurafenib, A549 cells were infected with the IAV strain SC35M and were subsequently treated with the p38 inhibitor SB202190, the JNK inhibitor SP600125 or with a combination of both in presence or absence of Vemurafenib (Figure 4E). Interestingly, even though the single inhibition of these MAPK pathways had more or less pronounced effects on viral progeny, a combination of the inhibitors led to a significant reduction in viral titers. Importantly, the inhibition of p38 and JNK in presence of Vemurafenib did not alter Vemurafenib-mediated interference with viral replication, emphasizing that Vemurafenib indeed acts antiviral via inactivating these MAPK pathways. Furthermore, the Vemurafenib-induced reduction in viral propagation was more pronounced compared to a combined p38 and JNK inhibition, suggesting the involvement of additional targets leading to efficient restriction of viral progeny by Vemurafenib.

Finally, we addressed the question as to whether the Vemurafenib-mediated inhibition of viral replication is based on a decelerated viral life cycle progression or whether Vemurafenib decreases the total number of infectious progeny viral particles produced over time. To discriminate between these two scenarios, A549 cells were infected with the IAV strains FPV or SC35M and were subsequently treated with Vemurafenib or DMSO to assess multi-cycle replication capability by standard plaque assays. Interestingly, Vemurafenib significantly limited viral replication of both viruses resulting in a prominent reduction of viral titers up to three orders of a magnitude compared to DMSO-treated control cells (Figure 4F).

In summary, Vemurafenib affects viral protein expression and delays nuclear export of progeny vRNPs leading to an efficient inhibition of viral replication at least in part by diminishing the activity of MAPK pathways. These results suggest a distinct interplay of Vemurafenib with various cellular signaling pathways resulting in a broad antiviral activity of the compound.

## Discussion

Seasonal IAV epidemics as well as recurrent pandemics reveal the need for effective novel treatments. A major drawback of the currently available antiviral inhibitors is the rapid adaptation and acquirement of resistances, which led to a paradigm change in finding new targets for antiviral approaches. An increasing attention is thus given to inhibitors of cellular signaling cascades that are required for efficient viral replication (Ludwig et al., 2003; Ludwig, 2009). One possible target is the Raf/MEK/ERK signaling cascade as it has important functions in viral replication (Ludwig et al., 2006). Inhibitors of MEK not only displayed low toxicity (Planz et al., 2001; Pleschka et al., 2001; Ludwig et al., 2004), but were also proven not to induce viral resistance (Ludwig et al., 2004). Interestingly, many different inhibitors of the Raf/MEK/ERK signaling cascade are developed and used in cancer therapy and therefore feature a well-characterized side-effect profile. In contrast to long-term cancer treatment, the repurposing of anti-cancer compounds for therapy of IAV infections is expected to result in less side-effects because of short-term use. In addition, side effects could even be further reduced by local application to the lung (Ludwig et al., 2006). Since Vemurafenib is already licensed for treatment of malignant melanoma (Kim et al., 2014) it is a promising candidate for use in anti-IAV treatment. The aim of this study was to analyze the impact of Vemurafenib on IAV replication in lung epithelial cells. Furthermore, the mechanistic background was analyzed, identifying Vemurafenib as a likely compound for antiviral intervention for the first time.

In the present study, a prominent antiviral activity of Vemurafenib against IAV was demonstrated for human and avian pathogenic virus subtypes *in vitro*. Particularly, the observation that highly pathogenic avian influenza viruses can be strongly diminished in their replication is important since these avian subtypes emerge with increasing incidence, can cause severe infections in humans and are not covered by seasonal vaccination. This highlights a broad mechanism of action against different IAV and indicates a potential use in case of appearance of new subtypes with possible pandemic risk. Of note, depending on the virus strain, Vemurafenib concentrations effective in limiting virus replication are comparable to observed inhibitory concentrations resulting in 50% inhibition of tumor cell growth ranging from 0.025 to 0.35 μM *in vitro* (Yang et al., 2012). Thus, concentrations needed for the treatment of influenza-infected patients with Vemurafenib especially considering local application to the lung should be within the range of clinically achievable concentrations.

Interestingly, the antiviral properties of Vemurafenib do not seem to be due to the effects of the compound on the Raf/MEK/ERK signaling cascade. In contrast to the described inhibition of the cascade in *BRAF*^*V600E*^-carrying cells, Vemurafenib was shown to paradoxically activate the ERK MAPK pathway in cells bearing oncogenic *RAS* or elevated upstream receptor signaling (Halaban et al., 2010; Hatzivassiliou et al., 2010; Heidorn et al., 2010; Joseph et al., 2010; Poulikakos et al., 2010). Consequently, the use of Vemurafenib as monotherapy against melanoma is controversially discussed since it was shown to promote cellular proliferation and to manifest clinically with progression of cutaneous squamous cell carcinoma or keratoacanthoma in some patients (Zhang et al., 2015). In accordance to that, IAV-infected oncogenic *RAS*-carrying A549 cells treated with Vemurafenib showed increased activation of the Raf/MEK/ERK signaling cascade. Nevertheless, there were no effects on cell proliferation or viability observable. Thus, short-term Vemurafenib treatment of influenza virus-infected patients exhibiting no abnormal lung physiology is not likely to induce strong side-effects based on a hyperactivated Raf/MEK/ERK pathway such as carcinoma, while showing efficient limitation of influenza replication. Furthermore, a second option would be the combined treatment of Vemurafenib along with MEK inhibitors to secure additional inhibition of Raf/MEK/ERK signaling, which should result in increased antiviral efficacy.

Our study shows that Vemurafenib strongly limits influenza virus replication by multi-target inhibition of IAV-induced activation of many signaling cascades especially of p38 and JNK MAPK pathways. Such a multifactorial mode of action was described earlier especially for different antiviral acting natural products, even though showing promising antiviral potential (Abdelwhab and Hafez, 2012). Due to the decreased activity of various virus-supporting pathways, identification of the main cellular actor(s) as well as the major point(s) of action in viral replication is quite challenging. For efficient replication, IAV depends on the tight regulation of apoptosis in infected cells to ensure efficient and timely nuclear vRNP export (Wurzer et al., 2003). The contribution of the ERK, p38 and JNK signaling cascades to apoptosis onset was already investigated in detail (Wada and Penninger, 2004) and in context of IAV infections, Akt, JNK and p38 were described to modulate apoptosis (Ludwig et al., 2006; Sumbayev and Yasinska, 2006; Zhirnov and Klenk, 2007; Hrincius et al., 2010; Marchant et al., 2010; Herold et al., 2012). Furthermore, Vemurafenib was already shown to influence apoptosis by dysregulation of MAPK signaling in melanoma cells (Kaplan et al., 2011; Gibney et al., 2013; Vin et al., 2013). In the present study, Vemurafenib treatment resulted in a decreased IAV-mediated expression of apoptosis-inducing cytokines, such as TRAIL. This might be primarily based on the Vemurafenib-induced inhibition of p38 MAPK activity since it was shown that TRAIL is expressed in a p38 MAPK-dependent manner in the context of highly pathogenic avian influenza virus infections (Börgeling et al., 2014). P38 MAPK as well as JNK were shown to be involved in IAV-mediated apoptosis regulation via the activator protein 1 (AP-1) (Ludwig et al., 2001, 2002). AP-1 and the specificity protein 1 (SP1) contribute to the expression and signaling of TRAIL, the latter by influencing death receptors DR4 and DR5 expression (Higuchi et al., 2004; Fassl et al., 2015). Additional experimental approaches are required to reveal whether inhibition of p38 MAPK activity is critical for Vemurafenib-mediated limitation of IAV-induced apoptosis or whether further signaling pathways affected by Vemurafenib are involved.

It was shown before, that activation of the Raf/MEK/ERK signaling cascade supports viral replication by enhancing the export of progeny vRNPs from the nucleus into the cytoplasm (Pleschka et al., 2001; Olschlager et al., 2004). While Vemurafenib hyperactivated this pathway, other MAPK cascades, such as p38 and JNK as well as the PI3K pathway were inhibited. Both, PI3K as well as p38 were also described to be important for the nuclear export of progeny vRNPs (Shin et al., 2007; Nencioni et al., 2009). Even JNK can be indirectly linked to nuclear vRNP export due to its function in apoptosis induction. Nonetheless, treatment with Vemurafenib only slightly delayed export of progeny vRNPs from the nucleus to the cytoplasm. This might be attributed to a compensatory effect of hyperactivated ERK-mediated active vRNP export and the inhibited apoptosis-mediated passive export. Thus, also here, combined treatment using Vemurafenib along with MEK inhibitors might be beneficial to limit ERK-mediated vRNP export from the nucleus, which might lead to a synergistic antiviral activity of the two compounds.

Interestingly, Vemurafenib treatment led to a decreased synthesis of viral proteins. Since viral transcription was not affected, this suggests a direct impact of Vemurafenib on the cellular translation machinery, which influenza viruses also depend on. One possible translation regulating kinase that is inhibited upon Vemurafenib treatment, is MK2. MK2 was shown to be activated in IAV infection and can directly interact with the repressor of the inhibitor of protein kinase R, finally resulting in PKR inhibition (Luig et al., 2010). PKR is activated by dsRNA and phosphorylates the eukaryotic initiation factor 2 (eIF2α), which results in the inhibition of protein translation, thereby limiting viral replication (Sadler and Williams, 2008). Interestingly, after influenza virus infection, MK2 was shown to be predominantly activated by p38 MAPK (Luig et al., 2010). Nevertheless, while knockdown of MK2 led to significantly reduced viral progeny, the effects of MAPK p38 inhibition are less pronounced and are controversially discussed (Nencioni et al., 2009; Luig et al., 2010; Börgeling et al., 2014). Of note, the antiviral action of Vemurafenib is more potent than a combination of p38 and JNK inhibitors indicating that Vemurafenib-induced restriction of viral replication involves more factors than p38 and JNK MAPKs only. The action of Vemurafenib seems to be rather based on the modulation of multiple cellular signaling pathways synergistically resulting in its potent antiviral activity.

While knowledge on the mechanisms of Vemurafenib-mediated inhibition of p38 and JNK is lacking so far, the paradoxical hyperactivation of the ERK pathway in B-Raf^WT^ cells that harbor upstream alterations such as oncogenic *RAS* or up-regulated receptor tyrosine kinases is driven by the formation of Raf dimers that lead to signaling through c-Raf and consequently to ERK pathway hyperactivation (Hatzivassiliou et al., 2010; Heidorn et al., 2010; Poulikakos et al., 2010). Interestingly, many different mechanisms of extensive crosstalk between the different MAPK pathways have been described including positive as well as negative feedback loops mainly mediated by phosphatases (Fey et al., 2012). Recently, it has been shown that even MEK1 and 2 can have different biological roles in regulated ERK activity (Ussar and Voss, 2004), with a non-canonical pathway of MKK3/MKK6/p38 activation induced by MEK2 only (Bouhamdan et al., 2015; Huth et al., 2016). Whether crosstalk of different MAPK pathways is responsible for Vemurafenib-induced inhibition of p38 and JNK or whether there is a more direct impact of Vemurafenib on these kinases needs to be further analyzed in the future.

In conclusion, the present study reveals for the first time that the clinically approved inhibitor Vemurafenib significantly impairs IAV infection. Furthermore, an overall inhibition of various cellular signaling pathways was detected in infected cells, concomitant with a hyperactivation of the ERK pathway. These findings demonstrate that Vemurafenib acts in a multifactorial manner, e.g. deregulation of viral protein expression and apoptosis modulation. While this multi-target mode of action may hamper identification of the underlying molecular principles, it also raises an intriguing perspective: A multi-target compound with diverse points of action in viral replication may even be less prone to the development of resistance compared to single-target drugs. Thus, fighting IAV infections with Vemurafenib might be a very promising and innovative approach for antiviral intervention.

## Author contributions

MH, TS, and YB: designed and performed the experiments, collected and analyzed the data, and wrote the manuscript; SL: analyzed the data and wrote the manuscript; CE: supervised the research, analyzed the data, and wrote the manuscript.

### Conflict of interest statement

The authors declare that the research was conducted in the absence of any commercial or financial relationships that could be construed as a potential conflict of interest.
