# Supplementary material for: Vemurafenib Limits Influenza A Virus Propagation by Targeting Multiple Signaling Pathways
Source: Front Microbiol. 2017 Dec 14;8:2426. doi: 10.3389/fmicb.2017.02426 (PMC5735105; doi:10.3389/fmicb.2017.02426)
Supplement: Supplementary file 1 [file DataSheet1.pdf]

Supplementary Material

Vemurafenib Limits Influenza A Virus Propagation by Targeting Multiple Signaling Pathways

Magdalena Holzberg, Yvonne Boergeling, Tobias Schräder, Stephan Ludwig, Christina Ehrhardt*

* **Correspondence:** Christina Ehrhardt: ehrhardc@uni-muenster.de

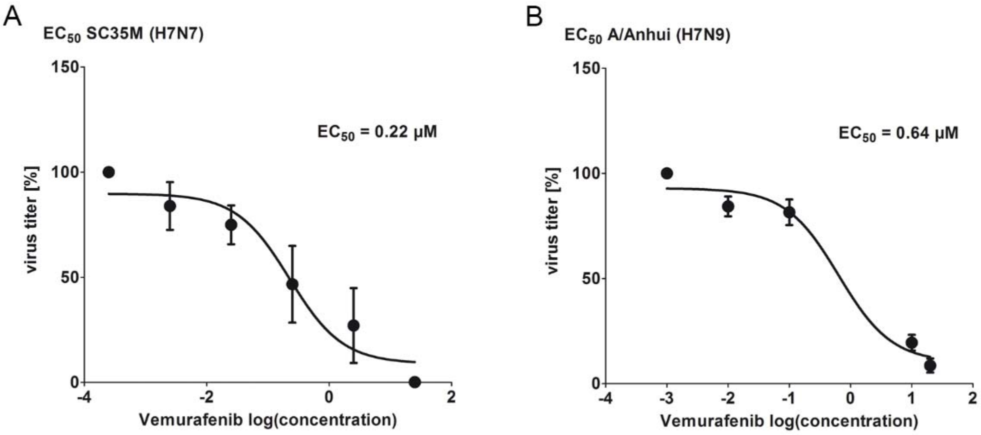

Figure S1. Determination of the effective concentration 50% (EC_{50}) of Vemurafenib. A549 cells were infected with 0.01 MOI **(A)** H7N7 (SC35M) or **(B)** H7N9 (A/Anhui). Cells were subsequently treated with different concentrations of Vemurafenib (0-25 µM). Progeny virus particles in the supernatant were measured by standard plaque assay 24 hpi. The EC_{50} values were calculated from three independent experiments with GraphPad Prism 5 software and depicted as mean (± SEM).

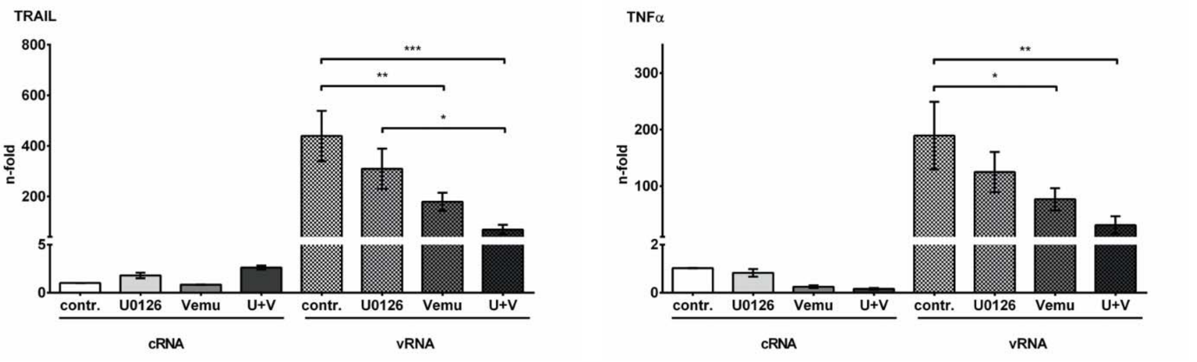

Figure S2. Vemurafenib limits IAV-mediated apoptosis-inducing cytokine expression independent of Raf/MEK/ERK signaling.

A549 cells were pre-incubated with U0126 (50 µM) for 90 minutes and Vemurafenib (25 µM) for 60 minutes or DMSO before transfection of 500 ng total RNA isolated from infected A549 cells (vRNA; FPV, MOI 5, 8 h). Total RNA from uninfected cells (cRNA) was used as control. The expression of TRAIL (left panel) and TNFα (right panel) mRNAs was measured by qRT-PCR 6 h post-transfection. Mean *n*-fold expression (± SEM) normalized to control (cRNA, DMSO) of three independent experiments is depicted. Statistical significance was evaluated by one-way ANOVA followed by Sidak's multiple comparisons test (**p* = 0.01-0.05; ***p* = 0.001-0.01; ****p* = 0.001-0.0001).

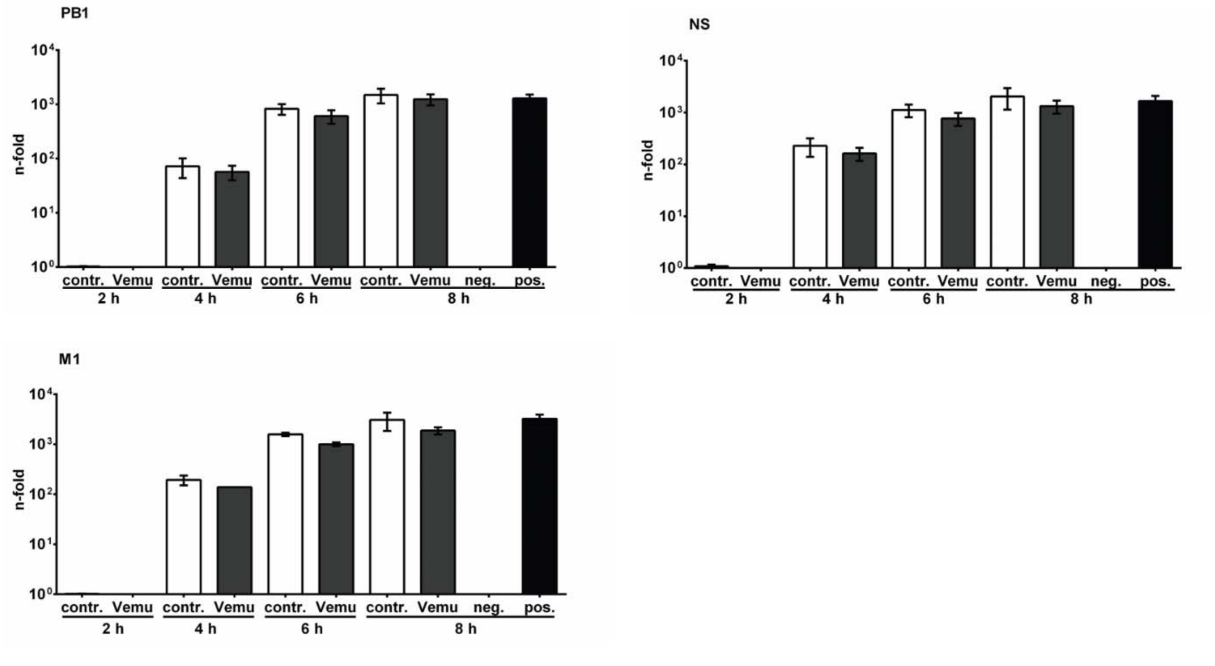

Figure S3. Vemurafenib has no impact on viral transcription.

A549 cells were infected with FPV (MOI 5) and afterwards treated with Vemurafenib (25 µM) or DMSO, respectively. Expression of PB1, NS or M1 m/cRNA was measured in triplicates by qRT-PCR at the indicated times. Untreated samples served as negative (uninfected) and positive (infected) control. Data are depicted as mean *n*-fold expression (± SEM) of three independent experiments normalized to negative control. Data were analyzed for statistical significance by Kruskal-Wallis test followed by Dunn's multiple comparisons test.
